# Effect of muscle atrophy on fracture healing: insights from a tibial musculoskeletal-finite element model

**DOI:** 10.1007/s10237-026-02101-6

**Published:** 2026-07-07

**Authors:** Qianjun Ding, Lunjian Li, Lihai Zhang

**Affiliations:** Department of Infrastructure Engineering, The University of Melbourne, Parkville, VIC 3010 Australia

**Keywords:** Muscle atrophy, Musculoskeletal modeling, Partial weight-bearing, Fracture healing, Finite element analysis

## Abstract

Patients with tibial fractures often suffer from muscle atrophy due to aging and postoperative immobility. However, much less is known about how atrophic muscle conditions interact with the microenvironment and influence fracture healing outcomes. To this end, this study developed an atrophy-adjusted tibial fracture musculoskeletal model to simulate physiological loadings on the fractured tibia associated with partial weight-bearing (PWB) walking rehabilitation, incorporating various degrees of muscle atrophy and clinically observed muscle deformation at fracture callus. An anatomically muscle-informed tibial fracture healing model, integrating muscle load distributions onto the insertion surface of the bone geometry, was then used to predict dynamic mesenchymal stem cell differentiations and deviatoric strains during gait. The effects of PWB% and rehabilitation walking speed on healing outcomes in patients with various levels of muscle atrophy were systematically evaluated. The results show that the muscle loadings and knee contact forces substantially decline with the increased level of muscle atrophy. Directly using musculoskeletal simulations without accounting for patient-specific muscle atrophy could overstate temporal fluctuations of healing trajectories, leading to overestimation in the risk of fracture non-union and unsuccessful angiogenesis. Besides, the tolerance of PWB% level and rehabilitation walking speed varies according to different levels of muscle atrophy. Our results can recommend PWB walking protocols to enhance endochondral ossification, while controlling the risk of vessel rupture. The study highlights the impact of muscle atrophy on the early healing process, aiming to assist physiotherapists and orthopedic surgeons in prescribing personalized rehabilitation protocols based on patient-specific muscle conditions.

**Supplementary Information:**

The online version contains supplementary material available at https://doi.org/10.1007/s10237-026-02101-6.

## Introduction

Tibial bone fractures are associated with a high economic burden for patients worldwide (Crespo et al. 2015). Beyond high surgical costs, 40% of patients are partially or fully unable to work (Schade et al. 2021). To reduce the impact of surgery on patients’ daily activities, partial weight-bearing (PWB) walking allows controlled lower limb motion in early recovery, promoting efficient callus and gait restoration (Abdalbary 2018). Previous clinical studies have shown that aging and postoperative movement loss could lead to various degrees of muscle atrophy (Gaston et al. 2005). Muscle strength can alter the loadings applied to the bone, leading to significant impacts of mechanical stimulation (Miramini et al. 2023). Appropriate mechanical stimulation could promote chondrocyte formation, encouraging endochondral ossification as an ideal healing pathway. Nevertheless, excessive movements can disrupt angiogenesis, elevating the risk of wound infections or delayed healing (Claes et al. 2002). Therefore, during the early healing stage, individualized PWB walking rehabilitation protocols need to be developed based on patient-specific muscle conditions.

Physiological loadings are key determinants of biological processes within the bone, which can be measured through in vivo experiments (Heinlein et al. 2009). Meanwhile, numerical simulation systems, such as OpenSim, have been widely used to overcome the challenges of clinical sampling. For example, *Trinler *et al*.* (Trinler et al. 2019) used 3D marker trajectories and ground reaction forces to estimate tibiofemoral loadings and muscle moment arms throughout the entire gait cycle. However, previous simulations that only focus on healthy individuals limit the applicability to patient-specific postoperative rehabilitation.

Finite element models (FEM) have been widely utilized in recent research to amalgamate mechanobiology theory with musculoskeletal simulation. Previous research integrated physiological loads at peak tibial muscle activity during gait into FEM to forecast tissue shear strain (Byrne et al. 2011; Ding et al. 2026a). Nonetheless, these simulations focused on static loads under healthy muscle conditions, whereas incorporating dynamic loads allows comparison in time-varying mechanical stimuli under various muscle conditions and discern critical loading stages.

To the author’s best knowledge, no known studies have addressed the impact of postoperative patient-specific muscle atrophy on healing outcomes. To this end, this study aims to explore the effects of walking speed and PWB% on dynamic tissue differentiation and angiogenesis at the fracture site, extending our previous musculoskeletal–finite element framework for distal radius rehabilitation (Li et al. 2025a). Specifically, we hypothesize that musculoskeletal loadings generated by atrophic muscles are disparate from those in healthy conditions. The findings would provide biomechanical insights for physiotherapists and orthopedic surgeons, facilitating the development of personalized rehabilitation protocols tailored to the early recovery needs of tibial fracture patients.

## Materials and methods

**Fig. 1:**
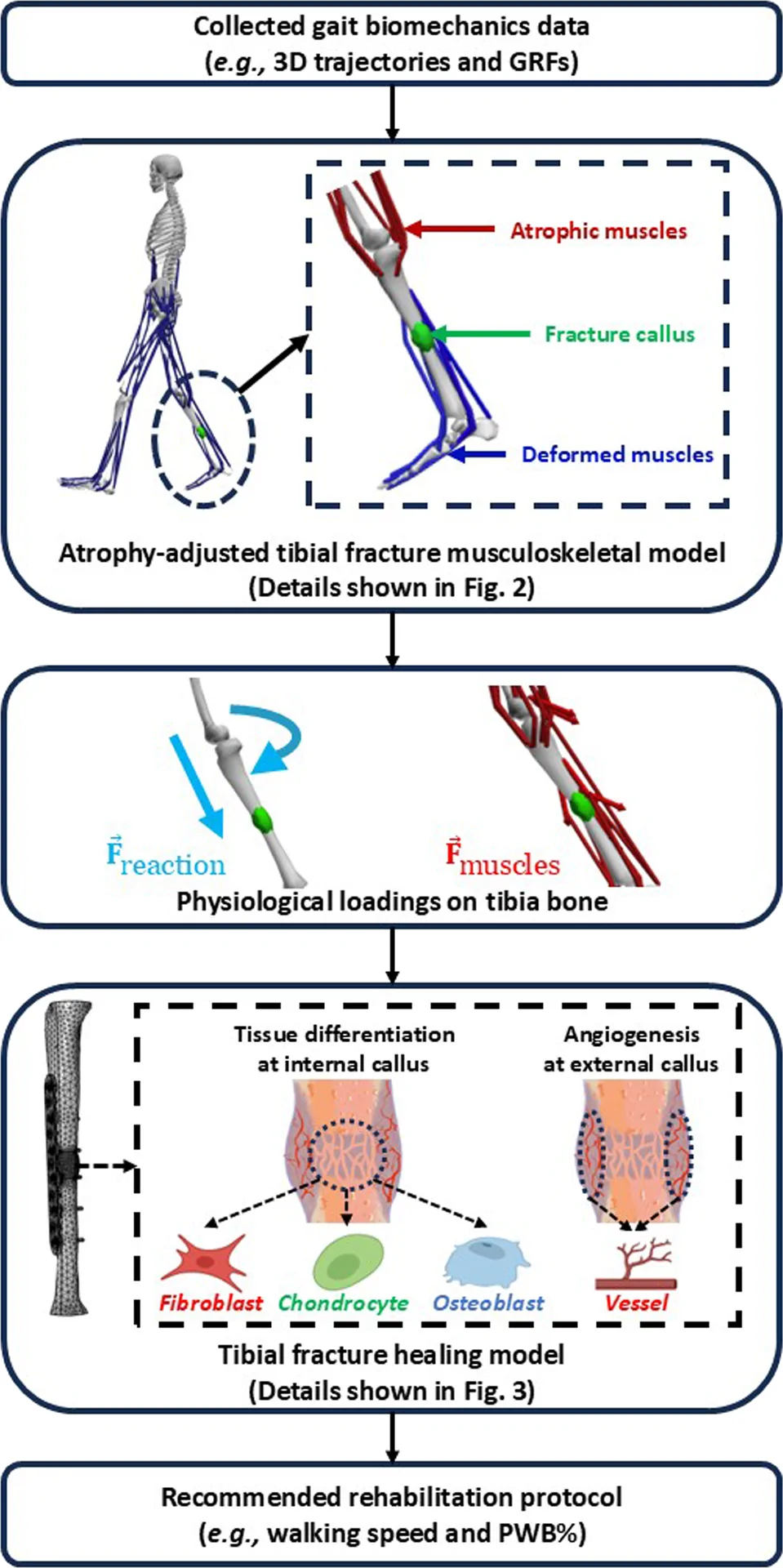
Schematic diagram of the overall computational framework

The overall computational framework used in this study is illustrated in Fig. 1. First, the biomechanical data were input into the developed atrophy-adjusted tibial fracture musculoskeletal model, which considers atrophy in lower extremity muscles during the early postoperative period. The physiological loadings on the tibia bone were computed and inserted into the enhanced tibial fracture healing model. Based on the predicted mechanical stimuli regulation of callus tissue and angiogenesis, the effects of muscle atrophy on healing outcomes under different rehabilitation protocols were discussed.

### Gait data preparation

This study uses gait biomechanics data from the previous experiment (Miramini et al. 2023). One healthy volunteer (age: 26 years; height: 178 cm; weight: 70 kg) was collected from the Biomotion Laboratory at the University of Melbourne. 3D gait trajectories with 24 retro-reflective markers and ground reaction forces (GRFs) were provided at 120 and 1080 Hz, respectively. To match the PWB rehabilitation protocols tolerated by patients with tibia fractures at the early healing stage (Braun et al. 2017; Kröger et al. 2022), the trials with walking velocities from 1–3 km/h were considered, and the first entire stance phase of the right foot was extracted per trial to simulate the complete dynamic force process of the right tibia. Ethical approval was granted by the University Human Research Ethics Committee, and the volunteer provided written informed consent before commencement.

### Atrophy-adjusted tibial fracture musculoskeletal model

**Fig. 2:**
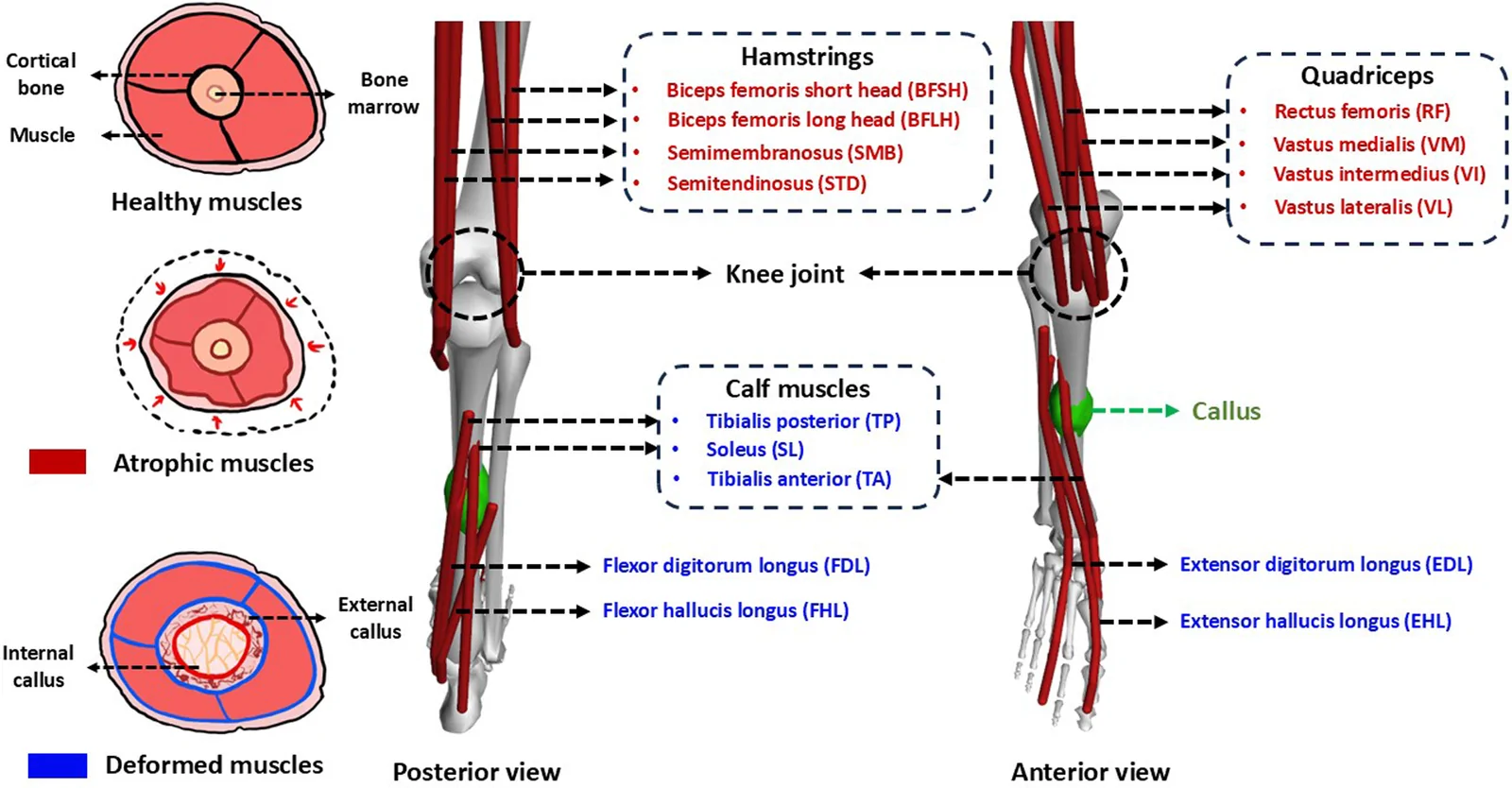
Bone geometries and muscle attachment of atrophy-adjusted tibial fracture musculoskeletal model

The atrophy-adjusted tibial fracture musculoskeletal model was developed based on the generic 3D gait musculoskeletal model in OpenSim v4.5 (Seth et al. 2018), consisting of 10 body segments, 23 degrees of freedom and 76 lower extremity muscles. Figure 2 demonstrates the relevant body joint and muscles attaching to the injured tibia, which influence the fracture site microenvironment. The quadriceps muscle group attaches to the anterior plateau of the tibia bone near the cortex, including the rectus femoris (RF), vastus medialis (VM), vastus intermedius (VI), and vastus lateralis (VL). The hamstring muscle group, including the biceps femoris short head (BFSH), biceps femoris long head (BFLH), semimembranosus (SMB) and semitendinosus (STD), attaches around the posterior tibial plateau. In addition, the flexor/extensor digitorum longus (FDL and EDL), flexor/extensor hallucis longus (FHL and EHL), along with tibialis posterior (TP), soleus (SL) and tibialis anterior (TA) of the calf muscle group, surround the tibia shaft.

During the early reparative phase (*i.e.,* week 3), the initial hematoma is gradually replaced by soft and hard callus tissue, stabilizing the fracture site. Concurrently, aging and immobility due to surgery can lead to muscle atrophy, whereas muscles around the callus can be deformed. These observations guided model modification to reflect loading during early rehabilitation. The hamstrings and quadriceps muscle groups are key contributors to knee reaction forces during gait. Previous longitudinal studies on postoperative (Gaston et al. 2005) and elderly cohorts (Kemmler et al. 2018), including 63 tibial fracture patients and 362 non-athletic men aged 19–91, respectively, have indicated that muscle atrophy can significantly reduce their peak isometric forces by up to 50%. Based on previous findings of comparable declines in quadriceps and hamstring strength (Stevens-Lapsley et al. 2010), a parametric analysis was performed by scaling muscle force-generating capacity from 0 to 50% reduction, in 10% increments, to represent varying degrees of postoperative muscle atrophy across patients. In addition, a cylindrical wrapping surface was added to simulate the appearance of soft callus originating from hematoma, matching the anatomically plausible muscle–tendon paths around the tibial shaft (*e.g.,* calf muscles, FDL, FHL, EDL and EHL) and bone geometry at the early fracture stage (Handsfield et al. 2014). The callus index of 1.1 and the working length of 30 mm were selected based on common callus geometry in patients with tibial fractures fixed using locking plates (Horn et al. 2011).

Upon configuring the muscle parameters and callus geometry of patients with tibial fractures, PWB levels of 10%, 20% and 30% were selected based on the possible weight-bearing range at the early stage of tibial fracture (Eickhoff et al. 2022; Zhang et al. 2017). Following the previous PWB simulations (Ding et al. 2026a), the gravity of the model and GRFs were reduced by 90%, 80% and 70% to match the walking rehabilitation condition using an anti-gravity treadmill. Dynamic joint angles were obtained using OpenSim’s inverse kinematics function, and were prescribed consistently across PWB% and atrophy levels to maintain controlled gait tasks. This controlled task design aims to isolate the muscle strength generation capacity to reduce the direct mechanical effects of internal musculoskeletal loadings and biomechanical stimulations at the fracture site, while preserving consistent external gait drive requirements. Individual muscle forces were computed using static optimization, which has been widely used in gait-driven musculoskeletal simulations to compute muscle forces and generate loading inputs for finite element analysis (Ding et al. 2026b; Guo et al. 2026). This algorithm considers joint moment constraints and maintains equilibrium between muscle forces and GRFs as

1 $$J=\sum_{m=1}^{n} {\left({a}_{m}\right)}^{2}$$

2 $$\sum_{m=1}^{n} \left[{a}_{m}f\left({F}_{m}^{0},{l}_{m},{v}_{m}\right)\right]{r}_{mj}={\tau}_{j}$$

where n represents the total number of muscles; ${a}_{m}$ represents the activation intensity of the muscle m at a particular timestamp; $f\left({F}_{m}^{0},{l}_{m},{v}_{m}\right)$ represents the force–length-velocity surface; ${F}_{m}^{0}$, ${l}_{m}$ and ${v}_{m}$ represent the maximum isometric force, length, and shortening velocity of the muscle m, respectively; ${r}_{mj}$ and ${\tau}_{j}$ represent the moment associated with the muscle m and the generalized joint torque toward the ${j}^{th}$ joint axis, respectively. Knee joint reaction forces at the tibia plateau were computed using OpenSim’s joint analysis tool. The time-dependent musculoskeletal outputs (*e.g.,* knee contact forces and individual muscle forces) were transferred as external boundary loadings to the tibial fracture healing model for subsequent mechanobiological simulations. This unidirectional loading transfer was used to isolate the biomechanical consequences of muscle strength under controlled rehabilitation gait conditions. The details of these force components under various muscle atrophy levels are shown in Supplementary Fig. 1.

### Tibial fracture healing model

The tibial fracture healing model was developed based on the previous validated model, which can predict early-stage healing outcomes under different loading conditions, consistent with relevant clinical findings (Miramini et al. 2023; Zhang et al. 2024, 2017). The bone microarchitecture properties, as demonstrated in Table 1, were applied to model the biphasic poroelastic material for analyzing the biomechanical microenvironment within the fracture callus, including tissue octahedral shear strain and interstitial fluid flow.

**Table 1:** Bone microarchitecture properties (Gardner et al. 2000; Lacroix and Prendergast 2002; Li et al. 2025b; Liu et al. 2021)

Properties	Fracture callus	Bone marrow	Cancellous bone	Cortical bone
Elastic modulus (MPa)	4.03	2	1300	17,000
Poisson’s ratio	0.17	0.17	0.3	0.3
Porosity	0.8	0.8	0.8	0.04
Permeability (${\mathrm{m}}^{4}/\mathrm{N}\mathrm{s}$)	${10}^{-14}$	${10}^{-14}$	${10}^{-11}$	${10}^{-17}$
Solid compression modulus (GPa)	2.3	2.3	2.3	1.39
Fluid compression modulus (GPa)	2.3	2.3	2.3	2.3

**Fig. 3:**
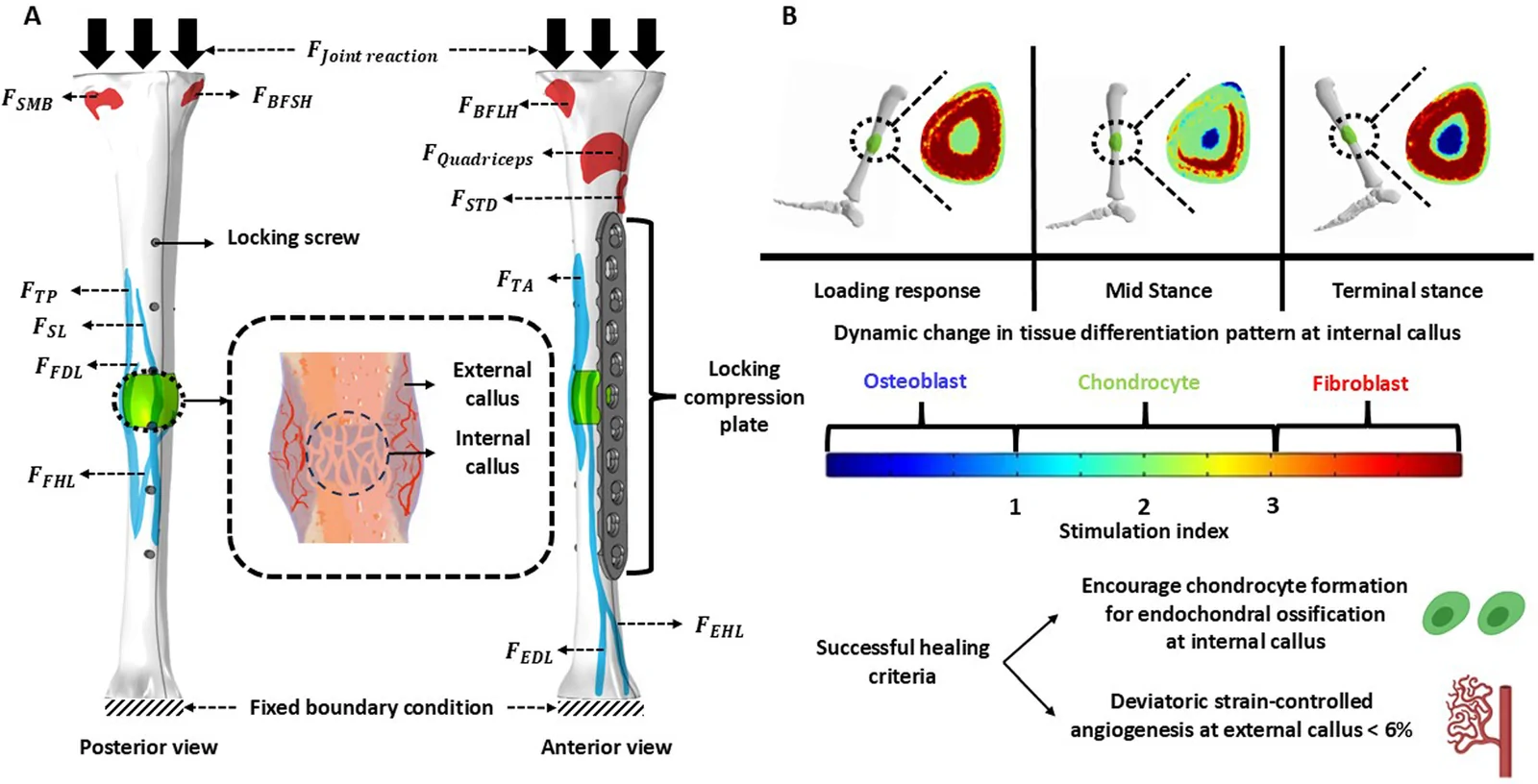
**A** Schematic diagram of the tibial fracture healing model; **B** Criteria of successful healing in the early stage of fracture healing based on dynamic changes of tissue differentiation during the stance phase (The mid-transverse sections of the internal callus are shown)

As shown in Fig. 3A, the tibial fracture was treated using a 4.5 mm-thick locking compression plate with six standard locking screws. All soft tissue boundaries were considered fully impermeable to fluid flow, and the interfaces between the soft tissues and fixator components were modeled as bonded solid-to-solid connections. The 3D model geometry was meshed and imported into finite element software COMSOL Multiphysics 6.2 (COMSOL Inc., MA, USA) for numerical simulations. To provide the most conservative strain-driven healing predictions, cortical bone, cancellous bone, marrow bone, fracture callus and locking plate fixation were meshed with the finest possible grids using 16,356, 9,553, 5,440, 22,717 and 17,733 s-order tetrahedral elements, respectively. Details of the mesh sensitivity analysis with peak and average principal strains are shown in Supplementary Fig. 2. This study further mapped muscle loading distributions to the insertion surfaces of the fractured tibia geometry to simulate stress and strain patterns under physiologically plausible muscle–bone interactions (Marie 2015). Muscle forces were obtained from OpenSim’s muscle–tendon actuators and then transferred to the fracture healing model as a distributed surface load applied over its anatomically defined insertion area using the COMSOL boundary load function. The distal tibia was fixed as a displacement constraint to prevent rigid-body motion of the isolated tibial FEM, consistent with previous studies on bone fracture and fixation simulations (Blažević et al. 2022; Wang et al. 2025). The proximal tibia was subjected to knee joint reaction forces. For each load, 3D force components derived from the musculoskeletal outputs were prescribed as time-dependent interpolation functions with a time interval of 0.01 s.

The mechano-microenvironment of the fracture callus can be simulated based on the porous media theory by the following governing equations (Zhang et al. 2008, 2009). Equations (3) and (4) solve for the effective stress of the solid matrix (${{\boldsymbol{\sigma}}}^{{\boldsymbol{e}}}$**)** and tissue stress tensor (${\boldsymbol{\sigma}}$), respectively.

3 $${{\boldsymbol{\sigma}}}^{\mathbf{e}}=\frac{1}{{J}^{S}}{F}^{s}\cdot 2\frac{\partial \left({W}^{s}\right)}{\partial {C}^{s}}\cdot {F}^{{s}^{T}}$$

4 $${\boldsymbol{\sigma}}=-p\mathbf{I}+{{\boldsymbol{\sigma}}}^{\mathbf{e}}$$

where ${J}^{S}$, ${F}^{s}$, ${W}^{s}$ and ${C}^{s}$ represent the deformation gradient, volume change, strain energy density and right Cauchy-Green deformation tensor of the solid matrix, respectively; p represents the incremental interstitial fluid pressure; $\mathbf{I}$ represents the identity matrix.

By assuming tissues under quasi-static conditions, the conservation of momentum and mass can be derived as Eqs. (5) and (6), respectively.

5 $$\nabla \cdot{\boldsymbol{\sigma}}=-\nabla p+\nabla \cdot {{\boldsymbol{\sigma}}}^{\mathbf{e}}=0$$

6 $$\nabla \cdot \left({\mathbf{v}}^{\mathbf{s}}-\mathbf{k}\nabla p\right)=0$$

where ${\mathbf{v}}^{\mathbf{s}}$ represents the solid phase velocity; $\mathbf{k}$ represents the tissue permeability tensor. By solving the governing equations, the tibial fracture healing model can compute the octahedral shear strain of the solid matrix (${\gamma }^{S}$), flow velocity of the interstitial fluid (${v}^{f}$) and tissue deviatoric strain% under PWB walking rehabilitations. Additionally**,** the time-dependent differentiation from mesenchymal stem cells to osteoblasts, chondrocytes and fibroblasts can then be simulated using mechano-regulation theory (Gardner et al. 2000; Prendergast et al. 2010), and their corresponding tissue formation can be formulated using a stimulus index (S), as shown in Eq. (7)

7 $$S=\frac{{\gamma }^{S}}{a}+\frac{{v}^{f}}{b}$$

where a= 0.0375 and b = 3 µm/s (Prendergast 1997).

Figure 3B illustrates the dynamic tissue differentiation pattern at the mid-transverse section of the fracture callus during the stance phase of gait (Prakash et al. 2018). Large (*i.e.,* S > 3), intermediate (*i.e.,* 1 < S < 3) and low (*i.e.,* S < 1) stimulus index leads to the formation of osteoblasts, chondrocytes and fibroblasts, respectively. To successfully heal the fracture callus, the endochondral ossification must be encouraged at the internal callus by maximizing chondrocyte formation, which enhances vascularization and mechanical stability (Kubiak et al. 2013). In this study, the percentage of chondrocyte formation under PWB walking was compared to that under 50% PWB static standing from the previous experiment (Miramini et al. 2023). Meanwhile, the deviatoric strain at the external callus was constrained to remain below the angiogenesis threshold of approximately 6%, as suggested by Claes et al. (Claes et al. 2011), to prevent non-union healing caused by ruptured blood vessels. The overall risk over 20% is defined as the inevitable failure of angiogenesis over the stance phase, according to the previous study (Liu et al. 2023).

## Results

### Simulation of musculoskeletal forces

**Fig. 4:**
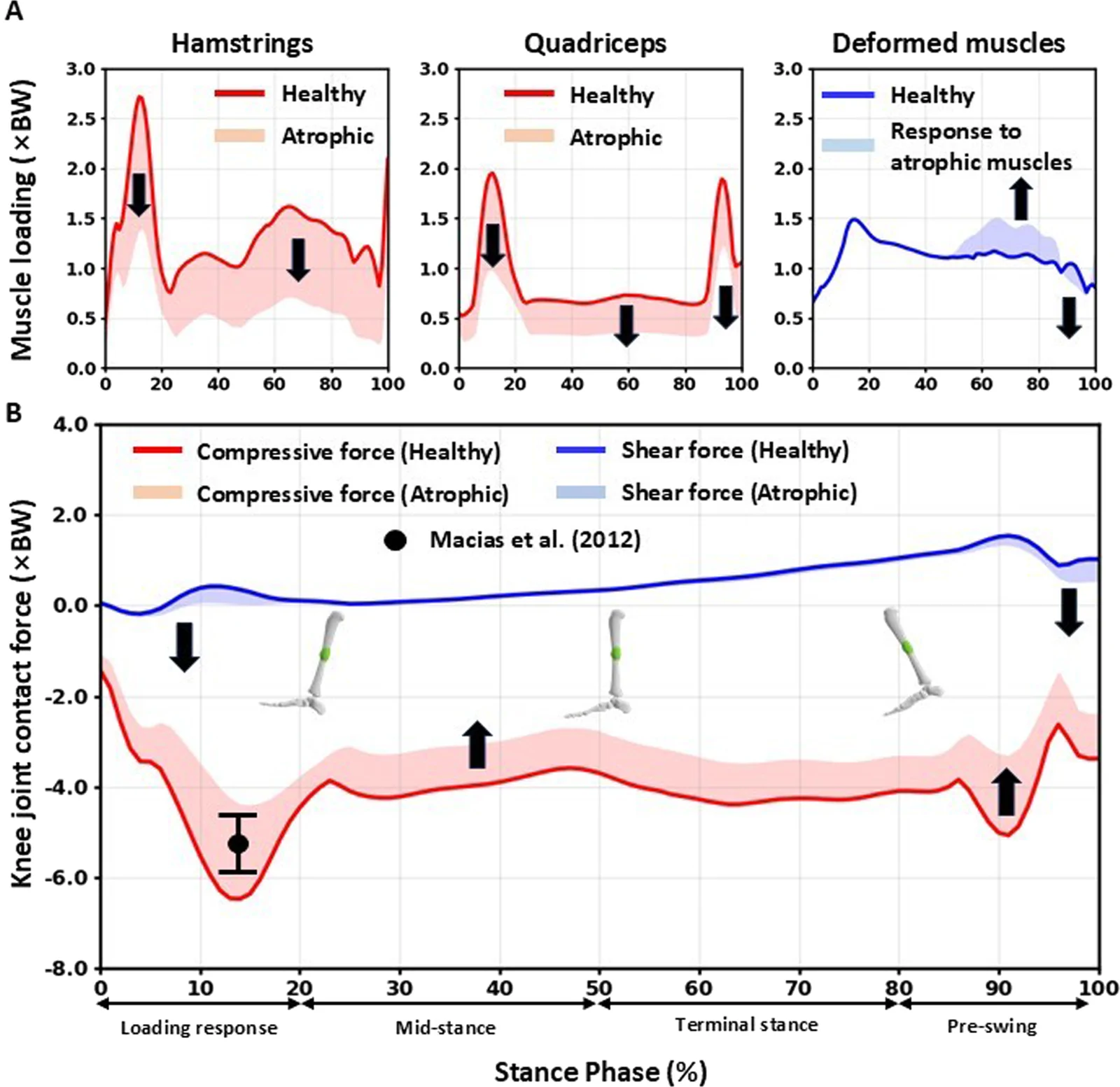
Comparison of **A** muscle loadings and **B** joint reaction forces between healthy and up to 50% atrophic conditions (PWB = 20%, speed = 2 km/h). The error margins represent the 95% confidence interval from experimental observations

Figure 4 illustrates the musculoskeletal forces simulated by the atrophy-adjusted tibial fracture musculoskeletal model under 20% PWB at 2 km/h. The maximum muscle activity on the tibia occurs before heel-off (*i.e.,* 15% stance phase). Muscle atrophy reduces peak loadings of hamstrings and quadriceps by up to 1.32 and 0.98 BW, respectively. In contrast, the deformed muscle group has the lowest overall loading contribution to the tibia. The generated forces in response to the atrophic muscles are increased by up to 31% during terminal stance and decreased by up to 24% during the pre-swing phase. The peak reserve actuator controls at the knee and ankle joints show a maximum peak reserve control at 2.3% and 0.4%, respectively. These low reserve levels indicate that the required joint torque is primarily supported by the simulated muscle and external loads, demonstrating a stable static optimization performance. More details about the peak reserve actuator controls during static optimization can be found in Supplementary Table 1.

The simulated muscle loadings were input into OpenSim’s joint analysis function to calculate knee contact forces. As shown in Fig. 4B, the knee contact loading patterns exhibit bimodal shapes, with peaks near the middle of the loading response and pre-swing phase. The maximum differences of shear and compressive forces occur at 11% and 14% stance phase, showing reductions up to 0.37 and 2.22 BW due to muscle atrophy.

### Prediction of fracture healing outcomes

**Fig. 5:**
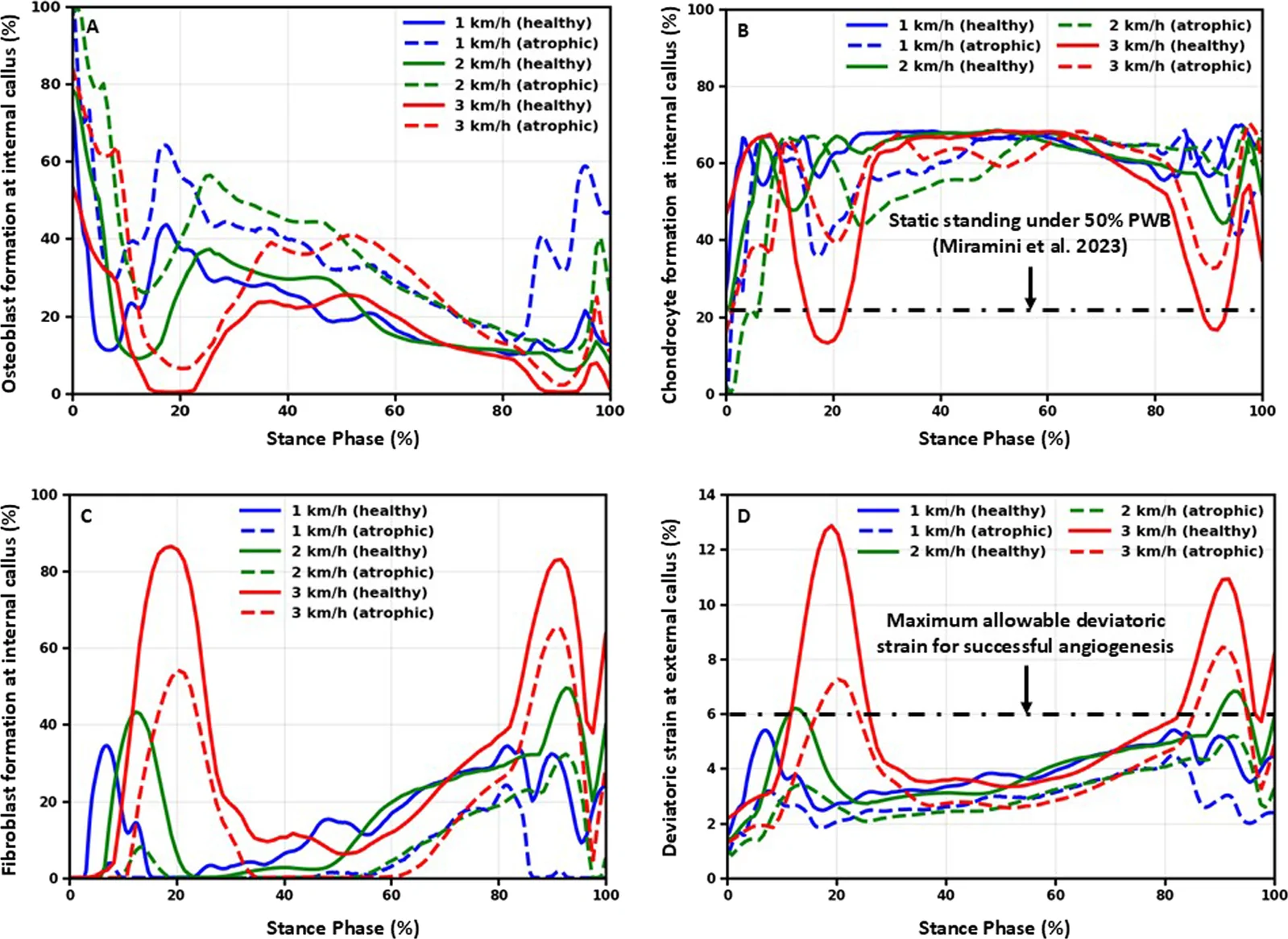
Predictions of healing outcomes under 20% PWB: **A** osteoblast; **B** chondrocyte; **C** fibroblast tissue formation at internal callus; and **D** deviatoric strain at external callus

Figure 5 highlights the temporal differences between healthy and 50% muscle atrophied limbs at various walking speeds under 20% PWB. Fibroblast formation exhibits an inverse relationship with osteoblasts at internal callus, while highly correlating with deviatoric strain at external callus. During the loading response, the lowest osteoblast formation at 1, 2 and 3 km/h corresponds to the maximum fibroblast formation, occurring at 5%, 10% and 20% stance phase, respectively. At the same speed, muscle atrophy leads to reductions in osteoblast formation, while discouraging fibroblast formation and reducing deviatoric strain throughout the gait. Based on previous observation (Miramini et al. 2023), the chondrocyte formation under 50% PWB static standing (*i.e.,* 21%) is used as a benchmark for internal callus. Besides, increased walking speed produces greater fluctuations in the chondrogenesis formation trajectories between healthy and atrophic muscle conditions, showing differences ranging from 51% at 1 km/h to 71% at 3 km/h. Furthermore, the deviatoric strain exhibits the greatest boost at a 3 km/h walking speed. Its peak value exceeds the maximum allowable angiogenesis benchmark (*i.e.,* 6%) by 6.9% and 2.4%, under healthy and atrophic muscle conditions, respectively.

**Fig. 6:**
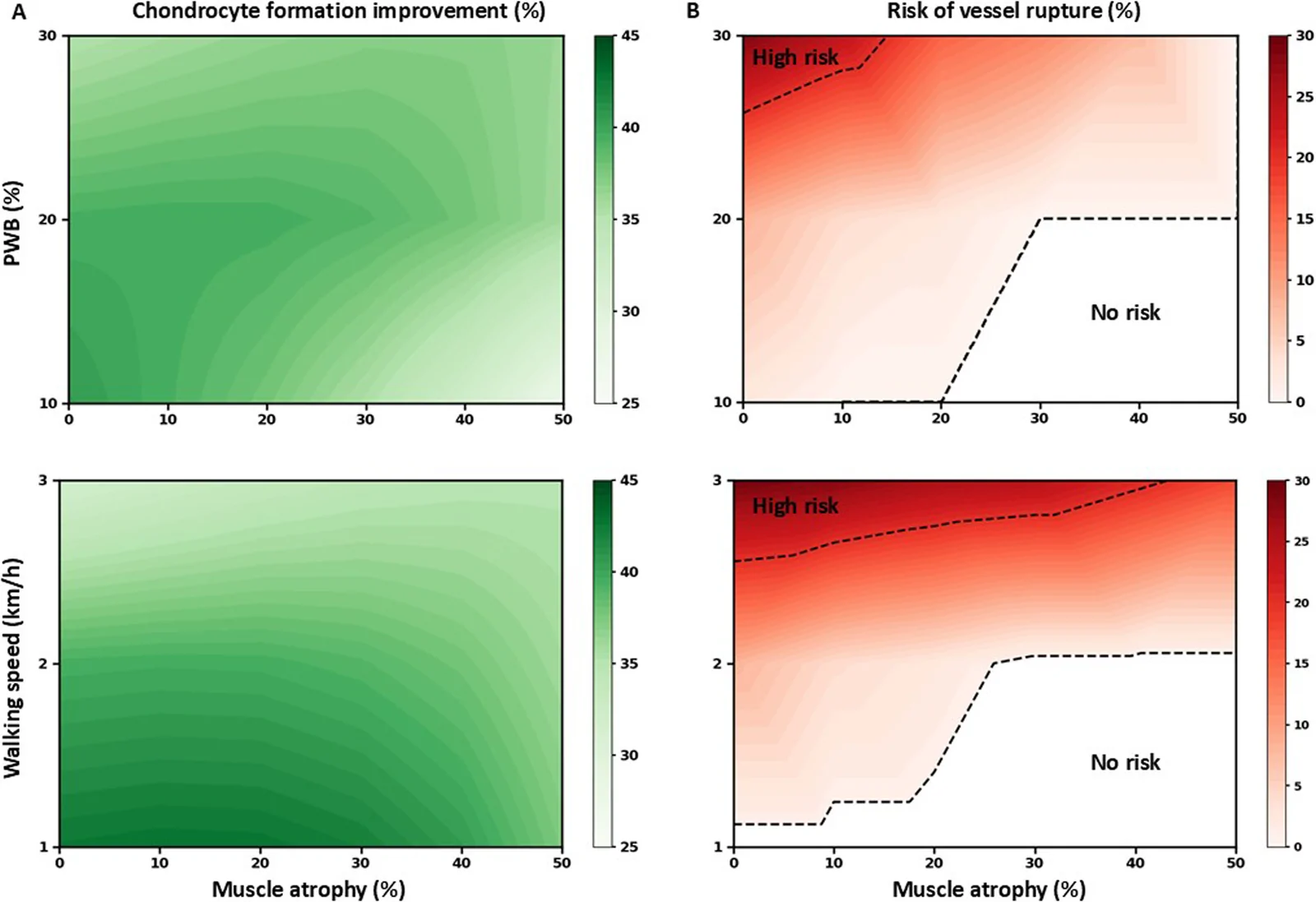
Effects of muscle atrophy under various levels of PWB (speed = 2 km/h) and walking speeds (PWB = 20%) on **A** chondrocyte formation improvement relative to 50% PWB static standing (*i.e.,* 21%); and **B** risk of vessel rupture relative to maximum allowable deviatoric strain (*i.e.,* 6%)

As shown in Fig. 6, the effects of muscle atrophy on healing outcomes are further examined under different PWB% and walking speeds. Under 10% PWB and 1 km/h, healthy muscle conditions show the most significant chondrocyte formation improvement, then declining monotonically with higher muscle atrophy, by 12.9% and 5.1%, respectively. Meanwhile, under 50% muscle atrophy, 30% PWB and a walking speed of 1 km/h significantly improve chondrocyte formation, reaching 36.4% and 37.6%, respectively.

As illustrated in Fig. 6B, the risk of vessel rupture was positively correlated with PWB% and speeds, while decreasing monotonically with greater muscle atrophy. The risk of vascular rupture remained constant at muscle atrophy levels from 0 to 20% with a speed of 2 km/h, reaching a maximum of 28.7%. Additionally, walking speed is more sensitive than PWB% in influencing angiogenesis, showing a maximum rise from 0 to 29.4% from 1 to 3 km/h under the healthy muscle case.

## Discussion

Based on the evidence on atrophy in lower limb muscles (Gaston et al. 2005; Kemmler et al. 2018) and fracture callus geometry (Horn et al. 2011), this study developed a patient-specific tibial fracture musculoskeletal model to compute physiologically plausible loadings on the fractured tibia during PWB walking. A biomechanical analysis on tissue differentiation and angiogenesis was performed using the tibial fracture healing model. The physiological loading estimation and mechanobiology-driven healing prediction are supported by previous experimental validations of musculoskeletal and FEM workflows (Kaneda et al. 2023; Miramini et al. 2016). The interpretation of the findings in this study should take into account the characteristics of unidirectional musculoskeletal-FEM coupling, whereas a fully coupled computational framework could further allow tissue-level biomechanical responses to influence kinematics adaptations (Ding et al. 2026a) or muscle recruitment (Henriksen et al. 2011). However, this study aims to reduce the confounding factors and examine the effects of muscle atrophy under controlled PWB walking conditions under rehabilitation, thus providing mechanobiological evidence to inform the design of patient-specific PWB protocols.

The simulated forces from atrophic muscles are lower than those of healthy muscles. This aligns with clinical observations that the patients naturally offload the injured side to minimize pain and prevent excessive mechanical stress at the fracture site (Larsen et al. 2017). This behavior reduces muscle activation, while deformed muscles show slightly increased mid-stance loadings compensating for weakened hamstrings and quadriceps. Meanwhile, callus geometry may induce passive stretching and fiber reorientation, increasing pennation angle and cross-sectional area, thus enhancing passive force outputs (Timmins et al. 2016). However, the simplified muscle representations from the musculoskeletal model should be considered when interpreting muscle loadings around the fracture site. More advanced musculoskeletal-FEM frameworks have incorporated 3D muscle modeling to improve anatomical fidelity (Li et al. 2019). These frameworks have incorporated complex muscle geometry (Modenese and Kohout 2020), fiber-dependent lever arms (Webb et al. 2014) and local muscle–bone interactions (Li 2021). Future studies should include additional geometric and tissue parameters (*e.g.,* callus index, osteon and muscle morphology) to further clarify the relationship between callus geometry and peri-callus muscle loadings.

Muscle loadings have significant contributions to joint reaction forces developed in the knee during walking, which are the primary contributors to the loadings applied on the fractured tibia (Sasaki and Neptune 2010). The simulated ranges of peak compressive forces with atrophic muscles are consistent with the observations from Macias et al. (Macias et al. 2012), who conducted an in vivo experiment on an 86-year-old adult walking using an upright lower-body compression treadmill exercise device. Although the validation was based on a single-subject in vivo, it primarily serves to demonstrate the physiological plausibility of the predicted loading patterns rather than subject-specific fidelity. Results indicate that knee compressive loadings are more susceptible to muscle atrophy than shear loadings. Compared to healthy muscles, atrophic musculature produces a flatter compressive force profile. For instance, decreased quadriceps strength can change the joint mechanics in knee extension, leading to fewer fluctuations before heel-off and after toe-off (Glass et al. 2013).

The compressive knee component generates the largest musculoskeletal loadings and aligns with dynamic changes in osteoblast and fibroblast formation, supported by previous findings (Li et al. 2023, 2025b). Excessive osteoblast formation during early healing can render the internal callus brittle and unstable, potentially promoting intramembranous ossification and increasing the risk of delayed healing or secondary injury (Marsell and Einhorn 2011). On the other hand, dominant fibroblasts due to unrestricted deviatoric strain can lead to sparse and disorganized blood vessels within the callus. This dysregulation limits nutrient delivery and osteoprogenitor recruitment, hindering inflammation resolution and increasing non-union risk (Claes et al. 2011). Additionally, previous computational simulations emphasized the role of muscle loadings on the biomechanical microenvironment at the fracture site during the early reparative phase (Li et al. 2023; Miramini et al. 2023). Based on our simulations, using loadings without considering atrophic muscles would overestimate the temporal fluctuations of healing trajectories and lead to inaccurate predictions of healing outcomes. At the same speed, muscle atrophy leads to reductions in osteoblast formation, while discouraging fibroblast formation and reducing deviatoric strain throughout the gait. By taking the case of 3 km/h walking speed as an example, the predictions under healthy muscle conditions show rapid declines in chondrocyte differentiation within the internal callus at the time of maximum muscle activities (*i.e.,* before heel-off and after toe-off). Meanwhile, compared to the predictions using 50% muscle atrophy, using healthy muscles results in consistently higher deviatoric strain at the external callus with a higher likelihood of exceeding the maximum allowable threshold for angiogenesis. Using musculoskeletal simulation in mechanobiological modeling without considering patient-specific muscle atrophy may overstate the risk of fracture non-union and unsuccessful angiogenesis, potentially misguiding clinical rehabilitation protocols.

Lack of movements (*e.g.,* static standing) could generate minimal mechanical stimulation at the fracture site, associated with insufficient chondrocyte formation even under 50% weight-bearing under the simulation. Our model predicts that slow walking (*e.g.,* 1 km/h) can maximize chondrocyte formation during the early healing stage, while the predicted optimal PWB% depends on degrees of muscle atrophy (*e.g.,* 10% PWB for healthy muscles and 30% PWB for 50% atrophy). Additionally, our simulations show that patients with different degrees of muscle atrophy have different angiogenic tolerances for weight-bearing and walking speed during rehabilitation. For instance, patients with lower muscle atrophy may warrant more cautious rehabilitation due to a high risk of vascular rupture. On the other hand, for patients with relatively high muscle atrophy, higher PWB% and moderate walking speeds ensure vascularization to remain within the modeled angiogenic tolerance at the external callus. This approach could also facilitate early muscle and gait recovery. However, in clinical practice, rehabilitation protocols should be further adjusted according to patient-specific pain tolerance.

### Limitations and future studies

This research has several limitations. Muscle deformation and bone properties (*e.g.,* callus and cortical density) were based on early postoperative clinical data from individuals. Muscle morphology and callus mineralization may vary with age, gender, BMI, and health conditions as healing progresses. In addition, the present computational framework couples the musculoskeletal and fracture healing FEM in a unidirectional manner. The predicted callus deformation and local biomechanical responses were not fed back to update gait kinematics, joint kinetics and muscle activations due to rehabilitation pain, which can further alter the absolute magnitudes and spatial distribution of mechanical stimulus at the fracture site. Therefore, the conclusion in this study aims to describe the comparative trends of biomechanical stimulation at the callus scale, rather than stress concentration at the attachment level. Future works should include larger cohorts to examine bone-muscle interactions at different healing stages and further integrate fully coupled framework approaches to capture biofeedback-driven gait adaptations.

The fracture healing model was established using a locking compression plate under standardized conditions (*e.g.,* callus index = 1.1, gap size = 3 mm) and conventional plate configurations (*e.g.,* bone–plate distance = 2 mm, working length = 30 mm). Tibial fracture exhibits varying degrees of severity and requires specific fixation techniques (*e.g.,* intramedullary nailing, Ilizarov fixators), which can significantly alter the local biomechanical environment. Comprehensive parametric studies are necessary to enhance generalizability.

This study concentrates on level-ground walking rehabilitation, but other walking activities, such as ramps and stairs, may exert distinct musculoskeletal loads on the tibia, hence affecting biomechanical stimulation at the fracture callus and rehabilitation procedures. Moreover, due to technological and ethical constraints, the data were obtained from a healthy young adult, while muscle atrophy and callus discomfort from tibial fracture might affect walking patterns. In addition, the deformed muscles attached near the fracture region were represented using standard OpenSim muscle–tendon actuators instead of explicit volumetric muscle geometries. As a result, the muscle belly deformation, muscle-fascia constraints and detailed load sharing around the muscle attachment areas cannot be fully captured. The simplification may influence the mechanical microenvironment of the fracture site surrounding the attached muscles. Future work should integrate atrophy-specific gait data or predictive simulations that allow re-optimization of kinematics (*e.g.,* OpenSim Moco) under muscle weakness to assess how the combined effects of muscle atrophy and adaptation alter joint loading patterns and stress distributions at the fracture site. Besides, the fidelity of local strain predictions can be further improved by incorporating 3D muscle geometry around the fracture callus.

## Conclusion

This study investigates the effects of atrophic muscles on tibial fracture healing by developing an atrophy-adjusted tibial fracture musculoskeletal model. The model can simulate musculoskeletal loadings on fracture of the tibia associated with PWB walking at the early stage of healing, incorporating various degrees of muscle atrophy and clinically observed muscle deformation. Additionally, an enhanced tibial fracture healing model incorporating anatomically informed planar muscle distributions was established to evaluate healing outcomes during dynamic rehabilitation. Some key findings are summarized as follows:

- Compared to healthy conditions, atrophic hamstrings and quadriceps substantially decline muscle loadings and knee contact forces applied to the fractured tibia. On the other hand, the loadings from the deformed muscle group exhibit compensatory increases, suggesting complex kinetics of the musculoskeletal system.
- Using musculoskeletal simulation in mechanobiological modeling without considering patient-specific muscle atrophy may overstate the likelihood of non-union and unsuccessful angiogenesis. Such overestimation becomes more pronounced with increased walking speed, potentially misleading rehabilitation protocol suggestions.
- Our simulations show that patients with different muscle status have different tolerances for weight-bearing and walking speeds during rehabilitation, suggesting that personalized rehabilitation strategies are worth exploring rather than directly recommending common clinical prescriptions.

## Supplementary Information

Below is the link to the electronic supplementary material.Supplementary file1 (DOCX 945 kb)

## Data Availability

No datasets were generated or analysed during the current study.
