# Supplementary material for: Effect of muscle atrophy on fracture healing: insights from a tibial musculoskeletal-finite element model
Source: Biomech Model Mechanobiol. 2026 Jul 7;25(4):78. doi: 10.1007/s10237-026-02101-6 (PMC13342410; doi:10.1007/s10237-026-02101-6)
Supplement: Supplementary file 1 — Supplementary file1 (DOCX 945 kb) [file 10237_2026_2101_MOESM1_ESM.docx]

**Supplementary Fig. 1: External boundary conditions to the tibial fracture healing model from (A) muscle and (B) joint reaction loading components (PWB = 20%, speed = 2km/h).**

**
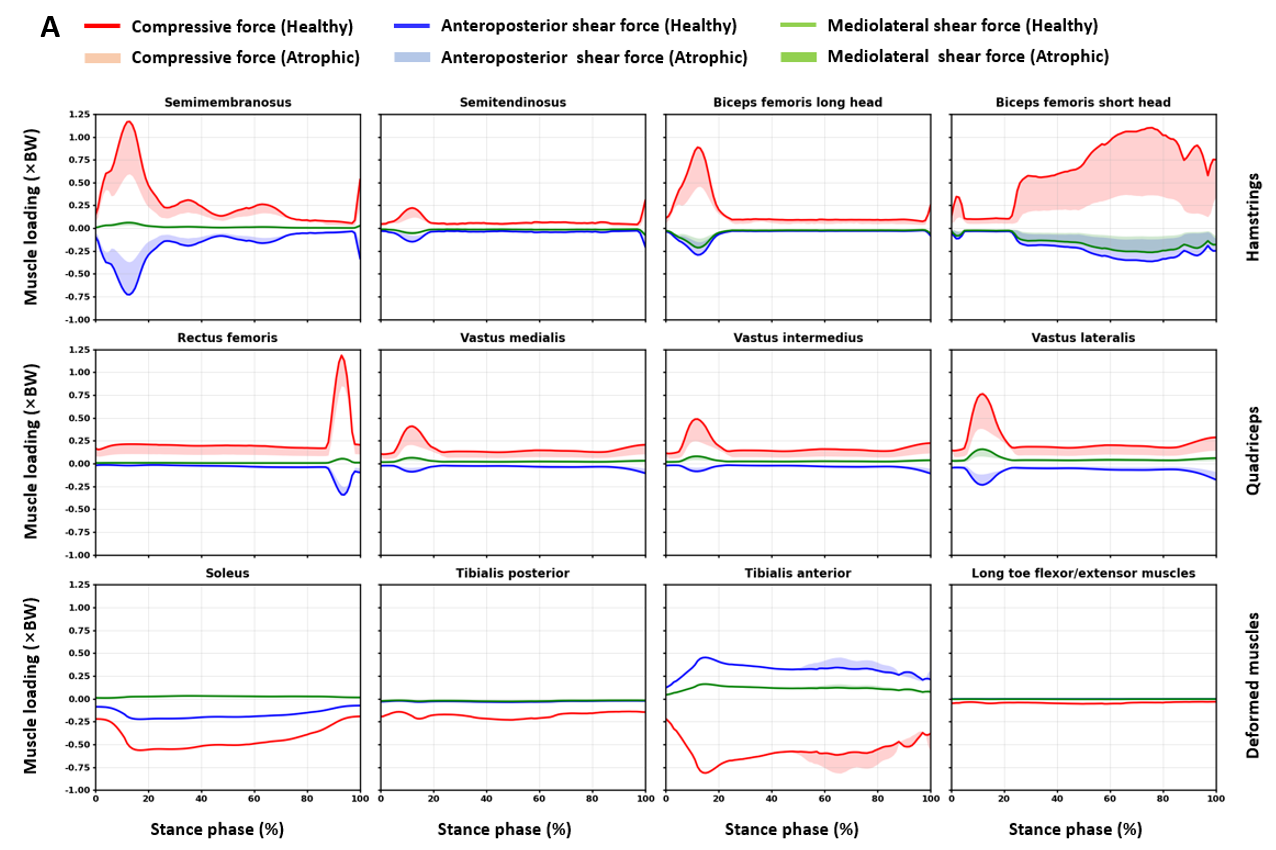
**


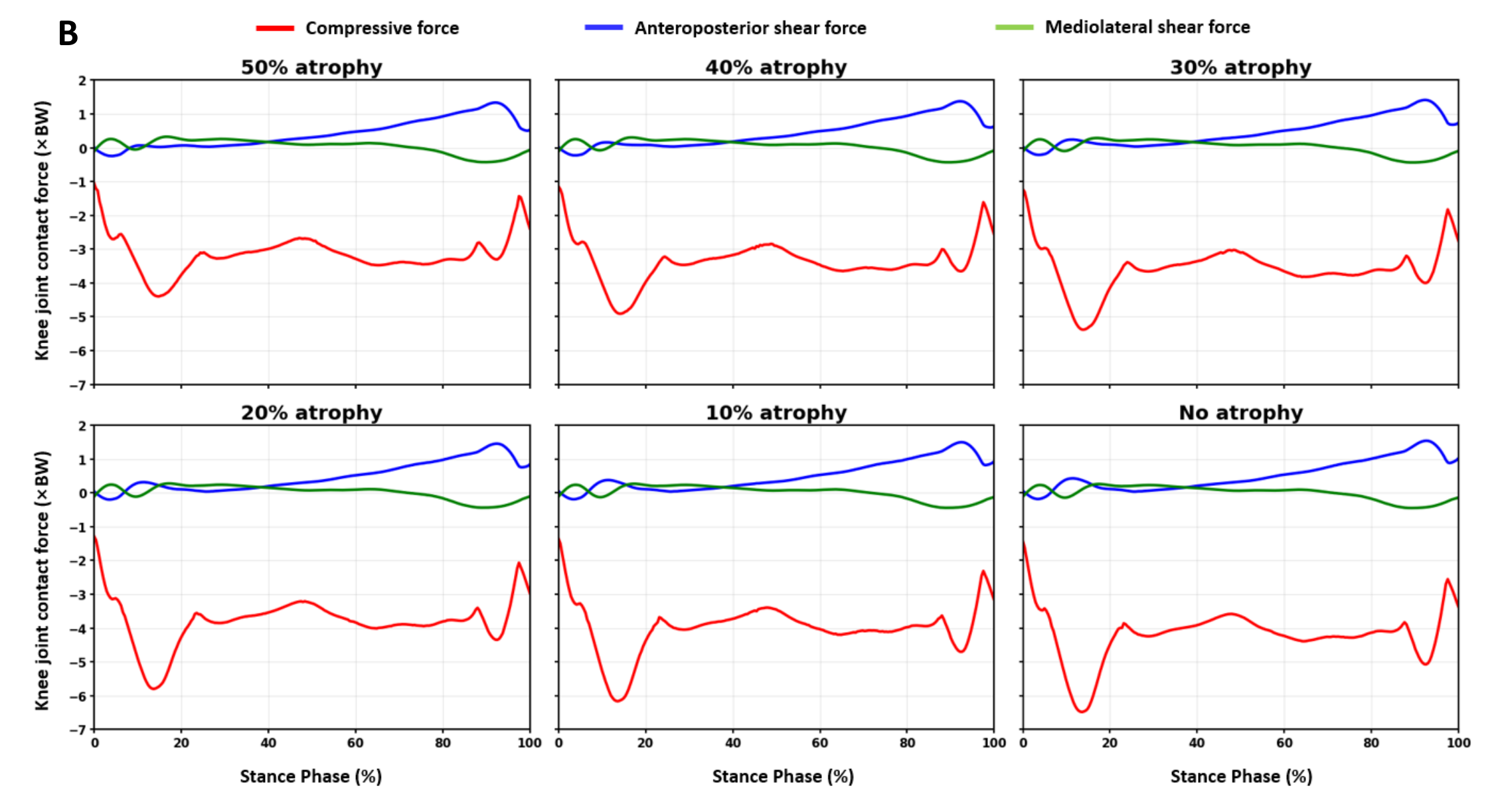


**Supplementary Fig. 2: Relative change in first principal strain (vs finest mesh).**


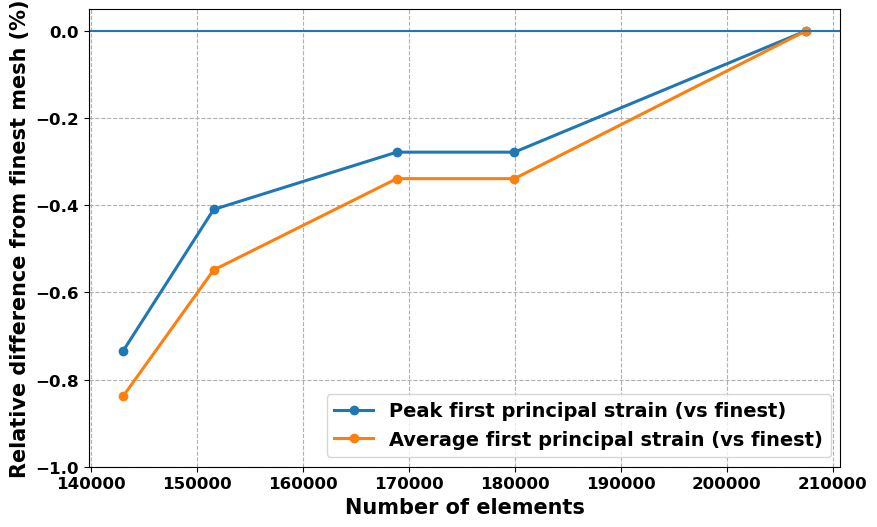


**Supplementary Table 1: Peak reserve activation of knee/ankle joints under various muscle atrophy levels and walking speeds at 20% PWB.**

|  | **No atrophy** | **10% atrophy** | **20% atrophy** | **30% atrophy** | **40% atrophy** | **50% atrophy** |
| --- | --- | --- | --- | --- | --- | --- |
| **1 km/h** | 0.5% / 0.3% | 0.6% / 0.3% | 0.6% / 0.3% | 0.6% / 0.3% | 0.7% / 0.3% | 0.7% / 0.3% |
| **2 km/h** | 0.6% / 0.3% | 0.6% / 0.3% | 0.7% / 0.3% | 0.7% / 0.3% | 0.8% / 0.4% | 0.9% / 0.4% |
| **3 km/h** | 0.9% / 0.4% | 1.1% / 0.4% | 1.2% / 0.4% | 1.5% / 0.4% | 1.8% / 0.4% | 2.3% / 0.4% |
